# Ectoparasite communities of small-bodied Malagasy primates: seasonal and socioecological influences on tick, mite and lice infestation of *Microcebus murinus* and *M. ravelobensis* in northwestern Madagascar

**DOI:** 10.1186/s13071-018-3034-y

**Published:** 2018-08-08

**Authors:** Annette Klein, Elke Zimmermann, Ute Radespiel, Frank Schaarschmidt, Andrea Springer, Christina Strube

**Affiliations:** 1 Institute for Parasitology, Centre for Infection Medicine, University of Veterinary Medicine Hannover, Bünteweg 17, 30559 Hanover, Germany; 2 Institute of Zoology, University of Veterinary Medicine Hannover, Bünteweg 17, 30559 Hanover, Germany; 3 Institute of Biostatistics, Leibniz University Hannover, Herrenhäuser Str. 2, 30419 Hanover, Germany

**Keywords:** Ectoparasites, Lemurs, *Haemaphysalis*, *Lemurpediculus*, Trombiculidae, Laelaptidae, Seasonality, Socioecology

## Abstract

**Background:**

Ectoparasitic infections are of particular interest for endangered wildlife, as ectoparasites are potential vectors for inter- and intraspecific pathogen transmission and may be indicators to assess the health status of endangered populations. Here, ectoparasite dynamics in sympatric populations of two Malagasy mouse lemur species, *Microcebus murinus* and *M. ravelobensis*, were investigated over an 11-month period. Furthermore, the animals’ body mass was determined as an indicator of body condition, reflecting seasonal and environmental challenges. Living in sympatry, the two study species experience the same environmental conditions, but show distinct differences in socioecology: *Microcebus murinus* sleeps in tree holes, either solitarily (males) or sometimes in groups (females only), whereas *M. ravelobensis* sleeps in mixed-sex groups in more open vegetation.

**Results:**

Both mouse lemur species hosted ticks (*Haemaphysalis* sp.), lice (*Lemurpediculus* sp.) and mites (*Trombiculidae* gen. sp. and *Laelaptidae* gen. sp.). Host species, as well as temporal variations (month and year), were identified as the main factors influencing infestation. Tick infestation peaked in the late dry season and was significantly more often observed in *M. murinus* (*P* = 0.011), while lice infestation was more likely in *M. ravelobensis* (*P* < 0.001) and showed a continuous increase over the course of the dry season. Genetic analyses identified *Lemurpediculus* sp. infesting both mouse lemur species. Ticks morphologically conform to *H. lemuris*, but genetic analysis showed a clear differentiation of the specimens collected in this study, suggesting a potentially new tick species. Host body mass decreased from the early to the late dry season, indicating nutritional stress during this period, which may render individuals more susceptible to parasitic infections.

**Conclusions:**

Seasonal differences and species-specific variations in sleeping site ecology in terms of sleeping site type and sociality were determined as key factors influencing ectoparasitism in *M. murinus* and *M. ravelobensis.* This needs to be taken into account when evaluating ectoparasite infestations at a given time point. The detection of the same parasite species on two closely related and sympatric host species furthermore indicates a potential pathway for disease transmission, not only within but also between lemur species.

**Electronic supplementary material:**

The online version of this article (10.1186/s13071-018-3034-y) contains supplementary material, which is available to authorized users.

## Background

For millions of years, parasites and hosts have coevolved and therefore usually exist in a delicate balance [1]. However, if parasite abundance becomes exceptionally high or the host faces additional external stressors, the impact of parasite infections can be severe [2–4]. Ectoparasites may affect individual body condition and fitness and thereby affect population health, but also need to be considered as potential vectors for inter- and intraspecific pathogen transmission. Non-human primates are of particular importance in this context, due to their phylogenetic proximity to humans [5]. Characterization of ectoparasite communities and identification of prevalence patterns in wildlife may shed light on wildlife population health and potential risks of disease emergence, especially in the light of habitat loss and increasing human encroachment into wildlife habitats. However, a variety of biotic and abiotic factors may affect parasite communities in wildlife. Therefore, it is essential to enhance our knowledge on these complex interrelations, before any conclusions regarding the effect of anthropogenic habitat disturbance can be drawn.

Socioecological factors may have a large impact on the ectoparasite communities of different host species. The results of experimental infestation of three different bird species with the tree-hole tick *Ixodes arboricola* provide support for the hypothesis that host selection may be limited predominantly by host ecology rather than species specialization [6]. The interaction of group size and parasite infections has been investigated across many taxa and is summarized by Rifkin et al. [7]. On the one hand, living in groups means, *inter alia*, a higher local host density and possibly higher interaction rates with conspecifics, thus increasing the risk of parasitism. On the other hand, group size may also have protective properties such as the encounter-dilution effect [8] or increased allogrooming rates, leading to more effective ectoparasite disposal [9].

Furthermore, sex-specific differences in ectoparasite infestation, either due to ecological determinants, such as differences in behavior or morphology, or due to different levels of the hormone testosterone and its immunosuppressive properties, are subject to controversial debate [10]. Hormonal differences have been discussed as a potential factor favoring ectoparasite infestation [11] and may trigger ectoparasite development [12]. In addition, abiotic factors, in particular temperature and humidity, are also known to affect ectoparasite development and survival rates. Seasonal variations in climatic conditions may therefore affect ectoparasite abundance and activity [13, 14].

Here, patterns of ectoparasitism were investigated in two closely related mouse lemur species endemic to Madagascar. Even though several studies have investigated ectoparasite communities of Malagasy lemurs in recent years [15–22], the drivers and dynamics of their ectoparasite load remain largely unknown. Mouse lemurs are the world’s smallest primates and show a high cryptic species diversity with 24 species currently described [23–26]. The grey mouse lemur, *Microcebus murinus*, and the golden-brown mouse lemur, *M. ravelobensis*, are of comparable small body size, nocturnal [27], arboreal and have a seasonally changing, diverse, omnivorous diet [28, 29]. They occur sympatrically in the strongly seasonal, dry deciduous forests of the Ankarafantsika National Park in northwestern Madagascar and are thus exposed to the same environmental conditions [27, 30]. Both species have a promiscuous mating system and show a seasonal reproductive activity with mating occurring towards the end of the dry season [31, 32]. However, despite the given similarities they show distinct differences in their socioecology regarding sleeping sites. *Microcebus murinus* spends the days in the protected wooden shelter of tree holes, and females, in particular, show high sleeping site fidelity [33]. In contrast, *M. ravelobensis* prefers a broader variety of sites in open vegetation, and shifts sleeping sites more frequently [34]. *Microcebus ravelobensis* is usually found in mixed-sex sleeping groups [35], whereas *M. murinus* shows sex-specific differences in sleeping behavior, with males mostly sleeping alone while females may be found sleeping in groups [33].

These differences in socioecology may influence parasite infection rates, life-cycle and distribution. Considering the fact that mouse lemurs spend over half of their lives in sleeping sites [36], it can be expected that the differences in sleeping site ecology, including the different degrees of sociality and corresponding variations in allogrooming rates, will be reflected in different patterns of ectoparasitism. We expected a higher frequency of ectoparasites transmitted by host contact (lice and mites) in the more gregarious *M. ravelobensis*, while parasite removal *via* allogrooming may reduce the frequency of temporary ectoparasites (ticks) in this species. Regarding sex, male mouse lemurs may be more susceptible to ectoparasite infestation than females due to the immunosuppressive properties of testosterone. Abiotic factors can also be expected to have a notable impact on ectoparasite infestations, in particular on temporary ectoparasites that only spend a limited time on the host and are more susceptible to environmental factors. While temperatures at the study site seem favorable year-round, precipitation and humidity are subjected to extreme variations in northwestern Madagascar and thus probably affect off-host ectoparasite survival and development. We therefore predicted a higher abundance of temporary ectoparasites during the hot and humid rainy season. Detailed analyses of the study populations’ body mass changes were included as a proxy of body condition and as such, as an indicator of environmental challenges, i.e. nutritional stress. Increasing food scarcity over the course of the dry season was predicted to impact host condition, rendering mouse lemurs more susceptible to parasitic infections. Overall, this study gives a comprehensive picture of the ectoparasite communities of *M. murinus* and *M. ravelobensis*, including a report on two mite species formerly not described in mouse lemurs, and a detailed description of a putative new *Haemaphysalis* species.

## Methods

### Study site

**Fig. 1:**
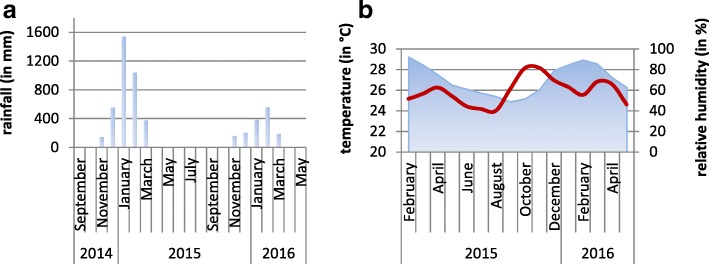
Climatic factors at the Ankarafantsika National Park (Ampijoroa Forest Station), Madagascar. **a** Precipitation in mm (Durrell Wildlife Preservation Fund, personal communication). **b** Temperature in °C (red line) and relative humidity in % (blue area) (Dr Hiroki Sato, Kyoto University, personal communication). For temperature and humidity, the monthly averages of hourly measurements are plotted

The study was conducted in the Ankarafantsika National Park, which is located in northwestern Madagascar, about 120 km southeast of Mahajanga. The climate in the dry deciduous forests of this region is characterized by a hot rainy season (November-April) and a relatively cooler dry season (May-October) (Fig. 1). Between April and November 2015 and from March to May 2016, 78 free ranging *M. murinus* (36 females, 42 males) and 100 *M. ravelobensis* (55 females, 45 males) were trapped and sampled in a designated mapped forest area of 30.6 ha called the Jardin Botanique A (JBA, 16°19'S, 46°48'E.).

The rainy season preceding the first field period had an exceptionally high total precipitation of 3688.6 mm; from November 2015 to April 2016 total rainfall was 1529 mm (Durrell Wildlife Preservation Fund, personal communication). Average yearly precipitation at the study site is 1562.5 mm [37]. Mean daily temperature during the study period varied between 17.57 °C (03.08.2015) and 30.05 °C (17.11.2015) (Dr Hiroki Sato, Kyoto University, personal communication).

### Mouse lemur trapping, weighing and sampling for ectoparasites

Mouse lemurs were trapped for 6 nights per month using Sherman live traps (Sherman Traps, Inc., Tallahassee, FL, USA). Ninety traps were installed in the late afternoon at each intersection of the rectangular trail system and baited with small pieces of banana. Traps were controlled in the early morning, or after about five hours in the offspring rearing season (March and April), respectively. Captured animals were individually marked subcutaneously with a small transponder (Trovan ID-100; Telinject®, Römerberg, Germany) that allows lifelong identification. Mouse lemurs were measured, weighed and sexed according to established methods [38] and macroscopically examined for ectoparasites. All animals were scanned systematically for ectoparasites with particularly thorough investigation of the head, ears and inner thighs at each capture. Collected arthropods were stored in 90% ethanol. Captured mouse lemurs were also checked for their reproductive status, with females being classified as “inactive” (no morphological changes), “cyclic” (reddening, swelling or opening of the vulva), “pregnant” (diagnosed by abdominal palpation and weight gain) or “lactating” (morphological changes in mammary glands, milk secretion), and males grouped in “testis large” (palpable testicles with a total width > 6 mm) or “testis small” (no testes palpable).

### Morphological and genetic identification of ectoparasites

#### Morphological identification

Upon return to Germany, all collected ectoparasite samples were investigated microscopically and 38 specimens [7 mites (3 Laelaptidae, 4 Trombiculidae), 11 lice, 20 ticks] were embedded in polyvinyl-lactophenol for further morphological measurements. Microscopic images and measurements were taken using a stereomicroscope (Stemi SV II, Zeiss, Jena, Germany) or Axiophote microscope (Carl Zeiss MicroImaging, Jena, Germany) equipped with a Colorview IIIu Camera, using cell^B Image Acquisition Software (version 3.1; Olympus Soft Imaging Solutions, Hamburg, Germany). Specimens damaged in relevant features were excluded from morphological measurements of the respective characteristic, leading to unequal sample sizes. Species identification was based on morphological characteristics if possible (ticks: [39–43]; lice: [44–46]; mites: [47–51]) and was complemented by an additional genetic analysis of a subset of samples.

#### Genetic genus/species identification

Six lice (4 from *M. murinus*, 2 from *M. ravelobensis*), 8 ticks (3 from *M. murinus*, 5 from *M. ravelobensis*) and 2 mites (1 Laelaptidae, 1 Trombiculidae, both from *M. murinus*) were used for genetic analyses. DNA was extracted from ticks individually homogenized with polystyrene pistils (Roth) using the DirectPCR® Lysis Reagent Cell (PEQLAB Biotechnology GmbH, Erlangen, Germany) following the manufacturer’s instructions. For selected tick specimens, a 760 bp fragment of the cytochrome *c* oxidase subunit 1 gene (*cox*1) was amplified using primers Cox1F und Cox1R [52] in a 25 μl reaction mixture containing 18.5 μl double-distilled water, 0.5 μl dNTPs (10 mM each), 1 μl for/rev primer (10 μM each), 2.5 μl 10× buffer, 0.5 μl Taq polymerase (5 Prime, Hilden, Germany) and 1 μl DNA template. Thermocycling conditions were as follows: 95 °C for 5 min followed by 40 cycles at 95 °C for 30 s, 55 °C for 1 min and 72 °C for 1 min, and final extension at 72 °C for 5 min. For a tick specimen a 240 bp fragment, and for lice specimens a 209 bp fragment of the *18S* rRNA gene was amplified using primers Ns1 and Ns2a [53] in a reaction setup corresponding to the recipe used for tick specimens. Thermocycling included an initial denaturation at 95 °C for 3 min, followed by 10 cycles at 95 °C for 30 s, 40 °C for 1 min and 72 °C for 1 min, and subsequently 40 cycles at 94 °C for 40 s, 48 °C for 1 min and 72 °C for 1 min, and final extension at 72 °C for 5 min. For two lice samples, additional *cox*1 sequences were amplified using primers L6625 and H7005 following the protocol of Light & Reed [54] but with adjustment of the elongation temperature to 72 °C. For the two mite samples, a 535 bp or 739 bp fragment of the *18S* rRNA gene, respectively, was amplified using primers 18Sfw and rev960 [55] in a reaction setup analogous to that for lice and tick specimens. Thermocycling conditions were: 94 °C for 3 min followed by 40 cycles at 94 °C for 1 min, 50 °C for 1 min and 72 °C for 1 min and final extension at 72 °C for 10 min. Amplified PCR products were loaded onto 1.5% agarose gels and visualized bands of the expected size were excised and purified using the Wizard® PCR Preps DNA Purification System (Promega GmbH, Mannheim, Germany). PCR products were Sanger-sequenced at the Seqlab Sequence Laboratories (Göttingen, Germany) or GATC Biotech (Cologne, Germany). For *18S* rRNA gene fragments of lice and tick specimens, additional sequencing primers (13+a and 13-a [53]) were used. Obtained nucleotide sequences were blasted against published sequences in NCBI GenBank and aligned using Clone Manager Professional Edition 9 (Scientific and Educational Software, Denver, CO, USA). Sequences were submitted to the GenBank database under the accession numbers MG132088-MG13294 and MG983747-MG983749.

#### Phylogenetic analyses

Phylogenetic analyses were performed for mite specimens as these could not be identified to the genus/species level by morphological characteristics or BLAST sequence comparison. The phylogenetic tree was constructed using the Maximum Likelihood method (Tamura-Nei model [56]) in MEGA7 software [57]. Bootstrap analyses [58] were performed with 1000 replicates.

### Data analyses

Taxon-specific presence-absence data were analyzed on the basis of all individual capture events by fitting generalized linear mixed effects models (GLMMs) with logit-link and binomial assumption for ticks, lice and both mite species, with the following fixed effects (predictive variables): species (*M. murinus*, *M. ravelobensis*), sex (male, female), seasonality (rainy season, dry season), reproductive status (females: inactive, cyclic, pregnant, lactating; males: testis large, testis small), age (juvenile, adult), weight and the temporal factors month and year (subsumed in seasons). When the different sampling time-points were included in statistical models on a monthly basis as influencing factors, parameters proved to be inestimable, leading to abstruse estimates and standard deviations. Sampling months were therefore grouped into seasons as follows: “late rainy season”, March until end of April; “early dry season”, first of May until July 15th; “late dry season”, July 16th until end of October; “early rainy season”, November. Repeated measurements were accounted for by including the grouping factor “animal ID” as a random-effect term. The influence of the study year was tested by comparing April-May 2015 to April-May 2016 as these were the only months sampled in both years. If no significant difference was determined (as was the case for tick infestation), data of both years was pooled by month for subsequent analysis, providing a larger dataset for these months. In case of lice and mites with significant differences between study years, the temporal factor was categorized as season-year, e.g. late rainy season 2015. Data from April 2015 had to be excluded from the GLMM for lice [leading to a drop out of 21 (1.6%) observations and a drop out of 3 individuals that were not captured again in later seasons], as the highly irregular distribution made it otherwise impossible to calculate a reliable model. Potential predictive variables were first tested one by one and successively added to the model, if influences were significant and/or improved the final model, based on the Akaike information criterion (AIC). The factor reproductive status, with the attributes “inactive”, “cyclic”, “pregnant” and “lactating” for females, and “testis large” or “testis small” for males, was tested separately in the final model, as the opposite sex is inherently excluded when the reproductive attributes are added to the model. Different models were compared *via* likelihood ratio tests (LRT). The predictive variable season was subjected to *post-hoc* analysis, computing all pairwise differences between seasons (in analogy to Tukey’s test) based on the parameters of the fitted GLMM , if identified as significant in the overall model. Statistics were performed in R version 3.1.2 (R Core Team 2014) using the packages *lme4* [59] and *multcomp* [60].

The animals’ body mass was analyzed as a response variable in a linear mixed effects model, including the fixed effects sex, species and their interaction. Juveniles and pregnant females were excluded from this analysis as their expected increase in body mass may obscure the weight development of the adult study population. Furthermore, the seasonal changes of weights were modelled as fixed effects with mean weight differences between seasons (early dry season, late dry season, early rainy season, late rainy season) and regression terms for mean change of weight per day within season, where regression slopes were allowed to differ between seasons. To account for repeated measures from the same individuals, random effects for differences between individuals, as well as differences between seasons within the same individuals were included, and finally a continuous autoregressive correlation structure (cAR(1)) was assumed for the residuals. A comparison of different random effect models based on AICc showed that model fit could not be improved by additionally assuming random slopes between individual or between seasons within individuals. The model was fitted using package *nlme_3.1-128* [61], AICc was computed using package *MuMIn_1.15.6* [62]. Based on the model fit the following *post-hoc* comparisons were performed (package *multcomp* [60]) including adjustments for multiple comparisons: pairwise differences of mean weights at the beginning of seasons were compared between seasons (in analogy to Tukey test), and regression slopes for weight change within seasons estimated and tested against a slope of 0. Subsets for *M. murinus* and *M. ravelobensis* were additionally analyzed separately for seasonal weight changes in the same way.

## Results

A total of 78 *M. murinus* (36 females, 42 males) and 100 *M. ravelobensis* (55 females, 45 males) were trapped and sampled for ectoparasites with high recapture rates leading to an overall sample size of 1306 separate capture events. The majority of animals (73.74%) contributed more than one data point to the final dataset. Individuals of both mouse lemur species were found to be infested with ticks, lice and two species of mites.

### Ticks and description of *Haemaphysalis* sp. “microcebi”

**Fig. 2:**
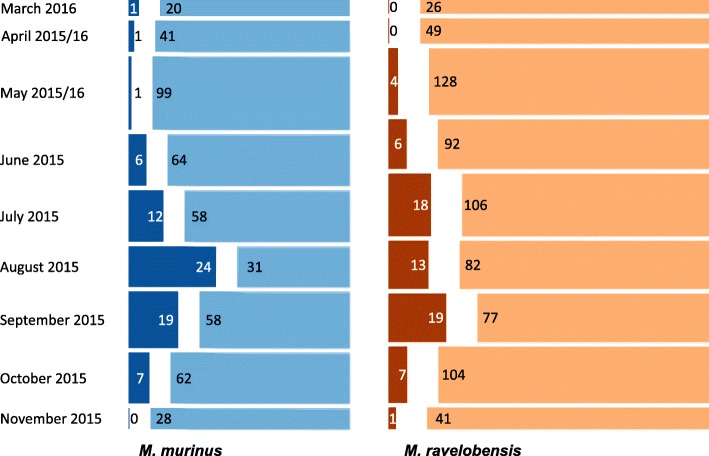
Monthly tick frequency for all *M. murinus* and *M. ravelobensis* captures. Tick-positive captures are shown in dark blue for *M. murinus* (*n* = 71) and dark brown for *M. ravelobensis* (*n* = 68), tick-negative captures are in light blue for *M. murinus* (*n* = 461) and light brown for *M. ravelobensis* (*n* = 705). Bar widths indicate sample size for the sampling month(s) and numbers indicate respective positive/negative captures

A total of 170 ticks were detected on captured mouse lemurs. Twenty-three tick larvae were recovered from 16 mouse lemurs (10 *M. murinus*, 6 *M. ravelobensis*) captured in the early months of the dry season (May, June and July 2015 and May 2016). Nymphs represented the majority of ticks (*n* = 145, 85.29%) infesting *M. murinus* (*n* = 73) and *M. ravelobensis* (*n* = 72) and were present from June to November 2015 with an infestation peak in August (Fig. 2). Only two adult male ticks were collected from two *M. murinus* in March and April 2016, and no adult female ticks were found on either of the two mouse lemurs. The GLMM revealed a significant influence of season and mouse lemur host species on tick infestation (Table 1). The highest tick incidence was recorded in the late dry season and the risk of infection was significantly higher for *M. murinus*. No significant differences were found between sexes and neither age nor reproductive status had a significant influence on tick infestation.

**Table 1:** Results of generalized linear mixed models (GLMM, logit-link, binomial assumption), and subsequent pairwise comparisons between seasons, respectively, for *Haemaphysalis* sp. “microcebi”, *Lemurpediculus* sp., Trombiculidae gen. sp. and Laelaptidae gen. sp.

Factor	Estimate	SE	*df*	*P*-value	Effect on parasite
*Haemaphysalis* sp. “microcebi”					
Species	-0.537	0.210	1	0.011^*^	*M. murinus* > *M. ravelobensis*
Sex	0.278	0.212	1	0.191	Male = female
Season			4		
EDS *vs* LRS	1.503	0.738		0.148	
LDS *vs* LRS	2.715	0.724		<0.001^*^	Late dry season > late rainy season
ERS *vs* LRS	-0.007	1.233		1.000	
LDS *vs* EDS	1.212	0.224		<0.001^*^	Late dry season > early dry season
ERS *vs* EDS	-1.509	1.025		0.412	
ERS *vs* LDS	-2.721	1.012		0.029^*^	Late dry season > early rainy season
*Lemurpediculus* sp.					
Species	1.446	0.219	1	<0.001^*^	*M. ravelobensis* > *M. murinus*
Sex	-0.594	0.382	1	0.120	Male = female
Season			5		
LDS15 *vs* EDS15	3.439	0.294		<0.001^*^	Late dry season 15 > early dry season 15
ERS15 *vs* EDS15	4.963	0.434		<0.001^*^	Early rainy season 15 > early dry season 15
LRS16 *vs* EDS15	2.897	0.353		<0.001^*^	Late rainy season 16 > early dry season 15
EDS16 *vs* EDS15	3.84	0.358		<0.001^*^	Early dry season 16 > early dry season 15
ERS15 *vs* LDS15	1.524	0.331		<0.001^*^	Early rainy season 15 > late dry season 15
LRS16 *vs* LDS15	-0.542	0.254		0.195	
EDS16 *vs* LDS15	0.401	0.253		0.489	
LRS16 *vs* ERS15	-2.067	0.398		<0.001^*^	Early rainy season 15 > late rainy season 16
EDS16 *vs* ERS15	-1.123	0.395		0.033^*^	Early rainy season 15 > early dry season 16
EDS16 *vs* LRS16	0.943	0.313		0.020^*^	Early dry season 16 > late rainy season 16
Reproductive status males	-1.204	0.277	1	<0.001^*^	Testis large > testis small
Trombiculidae gen. sp.					
Species	-1.375	0.484	1	0.005^*^	*M. murinus* > *M. ravelobensis*
Seasonality	3.177	0.655	1	<0.001^*^	Dry season > rainy season
Year	2.264	0.380	1	<0.001^*^	2016 > 2015
Sex	-0.259	0.480	1	0.590	Male = female
Laelaptidae gen. sp.					
Species	-2.592	0.808	1	0.001^*^	*M. murinus* > *M. ravelobensis*
Sex	0.932	0.621	1	0.133	Male = female

*Abbreviations*: *EDS* early dry season, *LDS* late dry season, *ERS* early rainy season, *LRS* late rainy season, *EDS15* early dry season 2015, *LDS15* late dry season 2015, *ERS15* early rainy season 2015, *LRS16* late rainy season 2016, *EDS16* early dry season 2016, *Estimate* difference between categories at the logit scale, *SE* standard error of the corresponding estimate based on the GLMM fit

^*^P < 0.05

Collected specimens were identified as haemaphysaline ticks and morphologically resembled the original description of *Haemaphysalis lemuris* by Hoogstraal [39]. Comparison of a 240 bp fragment of the *18S* rRNA gene (GenBank: MG983749) showed 100% identity with *H. punctata* (GenBank: Z74478), *H. longicornis* (GenBank: JQ346680), *H. concinna* (GenBank: KC511630), *H. sulcata* (GenBank: JX573126), *H. flava* (GenBank: JX573120) and *H. formosensis* (GenBank: JX573121), confirming the classification in the genus *Haemaphysalis*. However, even though comparison of the obtained 760 bp *cox*1 fragments (GenBank: MG132088-MG132092) showed 98 or 97% amino acid identity with *H. lemuris* sequences (GenBank: AFR33744 and AFR33745), substantial differences were observed regarding the respective nucleotide sequences, showing only 85% identity (GenBank: JX470177 and JX470178). This divergence suggests the presence of a separate species (hereinafter referred to as *Haemaphysalis* sp. “microcebi”).

**Fig. 3:**
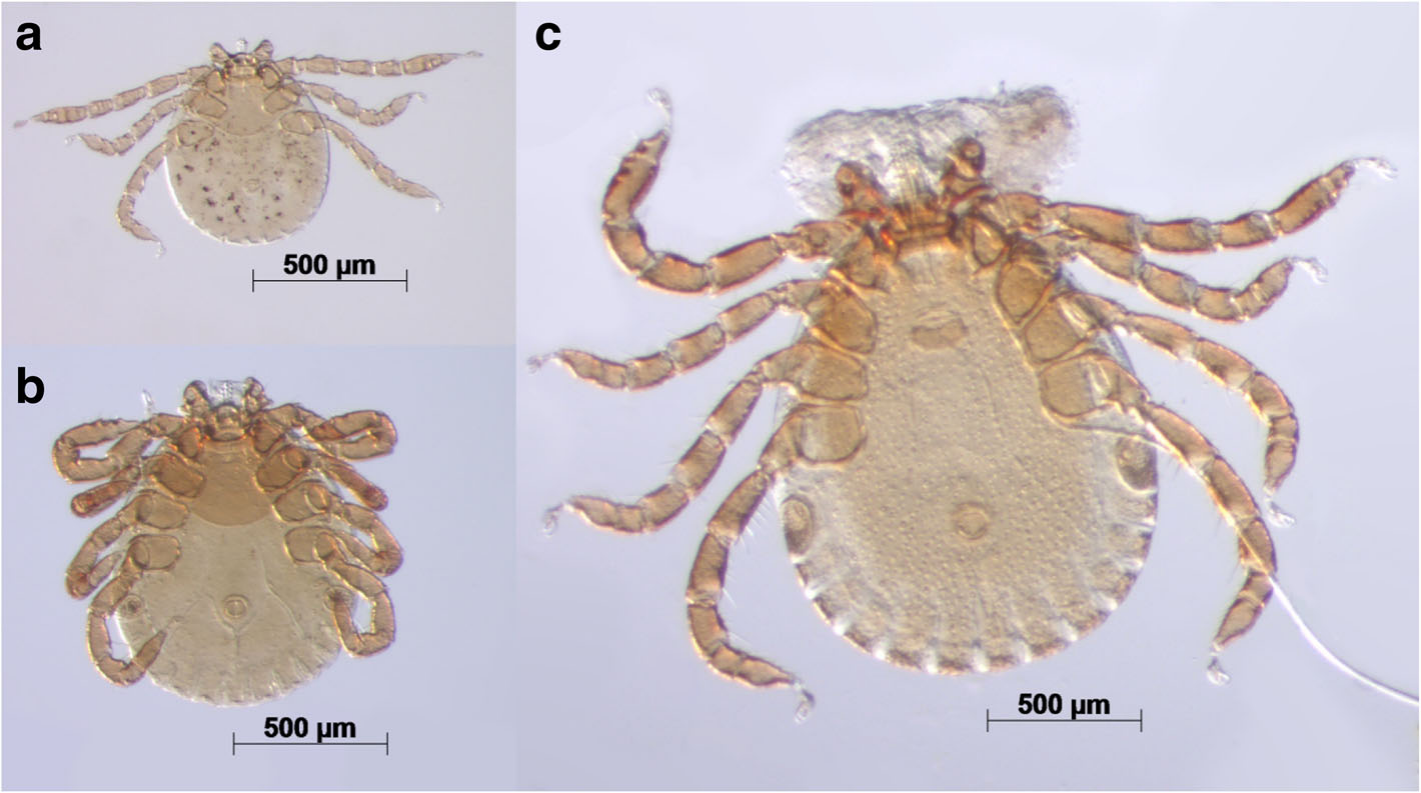
Different developmental stages of *Haemaphysalis* sp. “microcebi”. **a** Larva. **b** Nymph. **c** Adult male

Adult males and nymphs of *Haemaphysalis* sp. “microcebi” (Fig. 3) morphologically conform to the description of *H. lemuris*, with marked festoons, a triangular rounded spur on all coxae, flanked by a lateral bristle and a blunt spur on trochanter I to IV. Palpi are triangular with a convex basal margin and the hypostome shows a dentition pattern of 3/3 in male adults and 2/2 in nymphs, distinguishing the collected specimens from the morphologically very similar *H. simplex*. The two male adult specimens have a total length of 1753 and 1760 μm, respectively, with the scutum measuring 1251 and 1328 μm in length and 772 and 894 μm in width. The basis capituli is about half as long as wide (length: 112 and 123 μm, width: 229 and 238 μm). The hypostome measures 131 and 173 μm.

A total of 113 nymphs were measured with a mean total length of 1245 μm (range: 974–1808 μm). Total nymph length, unlike the other measures, did not correspond to a normal distribution, probably due to different feeding durations at the time of sample collection and consequently different levels of abdominal enlargement of collected ticks. The oval scutum measures on average 340 ± 26 μm in length (range: 282–388 μm; *n* = 64) and 500 ± 31 μm in width (range: 412–561 μm; *n* = 64), the basis capituli is 74 ± 15 μm long (range: 41–107 μm; *n* = 69) and 161 ± 13 μm wide (range: 119–190 μm; *n* = 72) and mean hypostome length is 114 ± 19 μm (range: 74–154 μm; *n* = 72). In the 23 recovered larvae of *Haemaphysalis* sp. “microcebi”, festoons are less pronounced, coxal spurs are sometimes missing or indistinct and dentition is 2/2 (Fig. 2). The average total length of larvae is 745 μm (range: 576–1172 μm; *n* = 20) and, as in nymphs, total length is not distributed normally. The mean scutal length is 189 ± 20 μm (range: 166–228 μm; *n* = 10), mean width 321 ± 17 μm (range: 289–347 μm; n = 9). The basis capituli measures on average 53 ± 8 μm in length (range: 44–68 μm; *n* = 13) and 102 ± 13 μm in width (range: 84–121 μm; *n* = 12), the hypostome is on average 74 ± 19 μm long (range: 43–108 μm; *n* = 13). Comparative measurements of the scutum, basis capituli, hypostome and length from tips of palpi to posterior scutal margin of *Haemaphysalis* sp. “microcebi” and 12 further Malagasy *Haemaphysalis* species are provided in Table 2.

**Table 2:** Descriptive measurements (in mm) of *Haemaphysalis* sp. “microcebi” and 12 further haemaphysaline species currently described in Madagascar. Developmental stages missing in this table have not yet been described

	Sample size	Total length (min-max)	Scutum		Basis capituli		Hypostome mean length	Dentition	Distinctive morphological feature
			Mean length	Mean width	Mean length	Mean width			
*Haemaphysalis* sp. “microcebi”									
Larva	20	0.58–1.17	0.19	0.32	0.05	0.10	0.07	2/2	All coxae with triangular spur, sometimes indistinct or missing in larvae
Nymph	113	0.97–1.81	0.34	0.50	0.07	0.16	0.11	2/2	
Adult ♂	2	1.75–1.76	1.29	0.83	0.12	0.23	0.15	3/3	
*H. lemuris* [34]									
Nymph	1	1.45	0.33	0.53	0.07	0.17	nd	nd	Coxae each with a short, broadly rounded spur; basal margin of palpi displaced at a 65° angle
Adult ♂	4	1.84–2.05	nd	nd	0.13	0.29	0.17	3/3	
Adult ♀	3	2.86–4	0.86	1	0.15	0.38	nd	nd	
*H. simplex* [34]									
Nymph	23	1–2	nd	nd	nd	nd	nd	3/3	Coxae each with a short spur (less pronounced in nymphs); basal margin of palpi straight or slightly convex
Adult ♂	110	1.98–2.64	nd	nd	0.11	0.25	0.17	4/4	
Adult ♀	17	2.3–2.6	0.92	0.92	0.15	0.38	nd	5/5	
*H. elongata* [34]									
Adult ♂	37	2.10–3.45	nd	nd	0.15	0.28	0.17	3/3	All coxae and trochanter with long spurs
Adult ♀	21	2.64	1.04	0.96	nd	nd	nd	3/3	
*H. subelongata* [34]									
Larva	43	nd	nd	nd	nd	nd	nd	nd	
Nymph	526	nd	nd	nd	nd	nd	nd	2/2	
Adult ♂	143	2.64–3.30	nd	nd	0.15	0.15	0.21	4/4	All coxae and trochanter with long narrow spurs; palpal lateral margin deeply concave
Adult ♀	17	nd	4	4.4	0.24	0.57	nd	4/4	
*H. theilerae* [34]									
Larva	4	1.32	0.26	0.30	nd	nd	nd	nd	
Nymph	12	1.32	nd	nd	nd	nd	nd	2/2	
Adult ♂	10	2.0–2.3	nd	nd	0.10	0.33	0.25	3/3	Palpi narrow, longer than greatest width; posterior groove in all coxae
Adult ♀	1	3.86	1.02	1.02	0.45	0.13	nd	3/3	
*H. obtusa* [34]									
Nymph	2	1.75	nd	nd	nd	nd	nd	2/2	
Adult ♂	52	1.62–1.86	nd	nd	0.15	0.25	0.12	4/4	Coxae each with a short pointed spur; palpal widest protrusion at mid-length
Adult ♀	42	1.79	0.75	0.80	0.10	0.33	nd	4/4	
*H. tiptoni* [34]									
Adult ♂	59	2.10–2.40	nd	nd	0.10	0.22	0.14	3/3	Coxa I with a needle-like spur
Adult ♀	21	2.3–2.7	0.92	0.79	0.13	0.35	nd	3/3	
*H. hoodi madagascariensis* [34]									
Adult ♂	1	2	nd	nd	nd	nd	nd	4/4	Coxae each with a rounded spine; palpal widest protrusion basally
Adult ♀	3	7.5	1.4	0.8	nd	nd	nd	4/4	
*H. fossae* [34]									
Adult ♂	17	1.8–2.1	nd	nd	0.18	0.30	0.16	4/4	Palpi 4.5 times as wide as long
Adult ♀	1	2.31	0.88	0.75	0.15	0.39	nd	nd	
*H. anoplos*									
Adult ♀	2	3.2	0.74	0.74	0.16	0.22	0.2	4/4	All coxae without spurs
*H. eupleres*									
Adult ♀	1	nd	1.1	0.9	0.22	0.43	nd	4/4	Palpi with spurs and groove
*H. nesomys*									
Adult ♂	1	1.8	1.58	1.03	0.14	0.18	0.1	3/3 to 4/4	Hypostome with irregular denticles

*Abbreviation*: *nd* not determined

### Lice

Based on morphological characteristics, collected lice specimens from both mouse lemur species were identified as *Lemurpediculus* sp., showing a strong resemblance with *Lemurpediculus verruculosus*, originally described by Ward [44] and previously reported as parasites of *Microcebus rufus* [45]. Comparison of six 209-bp *18S* rRNA sequences (4 from specimens ex *M. murinus* and 2 from specimens ex *M. ravelobensis)* with two published *L. verruculosus* sequences (GenBank: HM171410 and HM171409), showed a 100% identity, confirming the genus classification, but additional sequencing of fragments of the cytochrome *c* oxidase subunit 1 gene (GenBank: MG983747 and MG983748) revealed substantial differences to published *L. verruculosus* sequences (GenBank: HM171448 and HM171447). Collected specimens furthermore show variations in morphological characteristics within and between species, as well as to *Lemurpediculus madagascariensis*, a recently described new sucking louse species from *M. murinus* hosts of the Ankarafantsika National Park [46]. Lice collected in this study will therefore be conservatively addressed as *Lemurpediculus* sp.

**Fig. 4:**
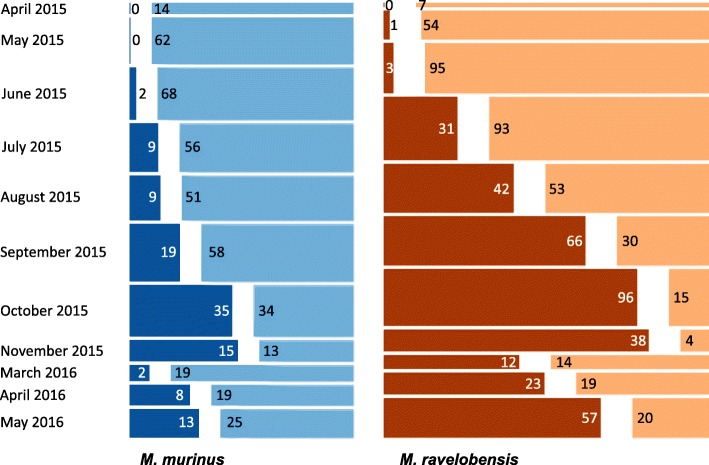
Monthly lice frequency for *M. murinus* and *M. ravelobensis*. Lice positive captures are shown in dark blue for *M. murinus* (*n* = 112) and dark brown for *M. ravelobensis* (*n* = 369), lice negative captures are in light blue for *M. murinus* (*n* = 419) and light brown for *M. ravelobensis* (*n* = 404). Bar widths indicate sample size for the sampling month(s) and numbers indicate respective positive/negative captures

Temporal parameters (month and year) influenced lice infestation significantly (Fig. 4). The risk of lice infestation significantly increased from the early dry to the early rainy season 2015 (*P* < 0.001), decreased towards the late rainy season 2016 (*P* < 0.001) and rose again until the end of the study period (*P* = 0.02) (Table 1). Lemur host species also proved to be of significant influence, with *M. ravelobensis* being significantly more often infected than *M. murinus* (*P* < 0.001). Host age did not have a significant influence on lice infestation. However, the presence of large testis during the period of reproductive activity from July to November, signaling hormonal activity, was associated with a higher likelihood of lice infestation (*P* = 0.009), although host sex as such did not significantly influence the lice infestation risk (Table 1).

### Mites

**Fig. 5:**
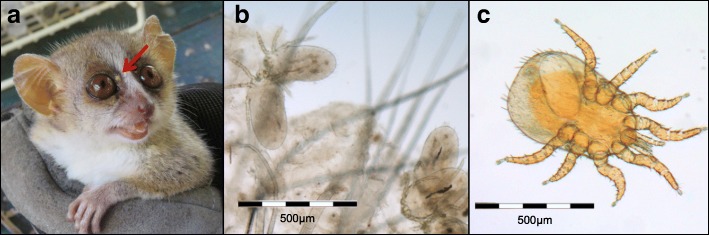
Mites found on *Microcebus* spp. **a** Characteristic accumulation of Trombiculidae gen. sp. near the eyes (shown is a male *M. murinus* host). **b** Microscopic view of Trombiculidae gen. sp. **c** Microscopic view of an adult female Laelaptidae gen. sp. individual (contains an egg)

**Fig. 6:**
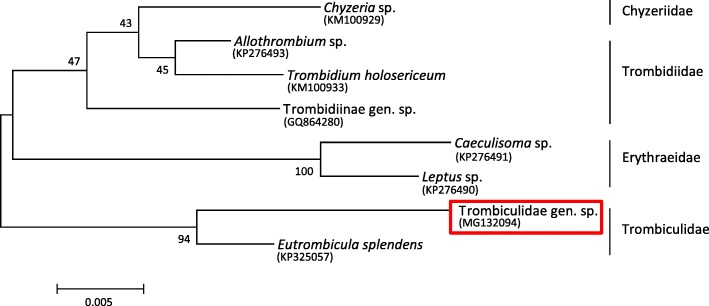
Phylogenetic tree of eight prostigmatic mites based on partial *18S* rRNA gene sequences using the Maximum Likelihood method. The percentage of replicate trees in which the associated species clustered together in the bootstrap test (1000 replicates) is shown next to the branches. The sequence of the trombiculid mite of the present study is framed in red

Two different species of mites (Trombiculidae gen. sp. and Laelaptidae gen. sp.) were collected from *M. murinus* and *M. ravelobensis* (Fig. 5). Trombiculidae gen. sp. were isolated from scrapings of scurfy, crusty skin alterations, particularly around the eyes and snout, and morphologically identified as chigger larvae. Sequence comparison of a 535-bp *18S* rRNA gene fragment with published sequences revealed 98% identity with *Eutrombicula splendens* (GenBank: KP325057), confirming classification in the family Trombiculidae (Fig. 6). Characteristic skin conditions caused by larvae of Trombiculidae gen. sp. were significantly more often observed in *M. murinus*. Furthermore, significant differences were apparent between the different sampling seasons and years (Table 1). The risk of Trombiculidae infestation was higher in the dry than in the rainy season (*P* < 0.001) and higher in 2016 than in 2015 (*P* < 0.001). Neither host sex, nor reproductive status had a significant effect on Trombiculidae infestations.

**Fig. 7:**
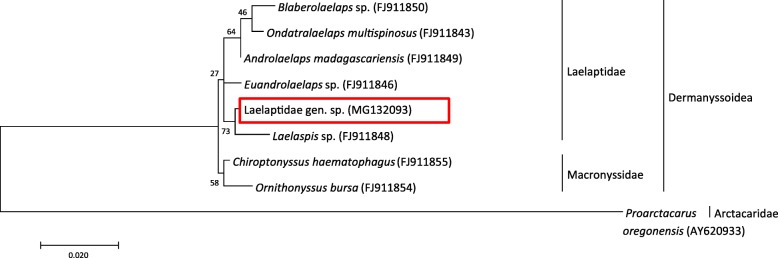
Phylogenetic tree of eight dermanyssoid mites based on partial *18S* rRNA gene sequences using the maximum likelihood method. The percentage of replicate trees in which the associated species clustered together in the bootstrap test (1000 replicates) is shown next to the branches. The sequence of the laelaptid mite of the present study is framed in red. The sequence of *Proarctacarus oregonensis* was used as an outgroup

The second mite species was only observed on 19 sampling occasions and significantly more often in *M. murinus* (*n* = 17) than in *M. ravelobensis* (*n* = 2) (*P* = 0.001) (Table 1). Genetic analysis of a 739-bp *18S* rRNA gene fragment placed the collected specimens in the mite family Laelaptidae, showing 99% identity with *Androlaelaps madagascariensis* (GenBank: FJ911849) (Fig. 7), but morphological characteristics did not match any of the species previously described in Madagascar. No significant influence on Laelaptidae gen. sp. infestations was found for host sex. Regarding seasonal differences, the frequency of Laelaptidae gen. sp. occurrence was too low to perform reliable statistical analyses.

### Body mass

**Fig. 8:**
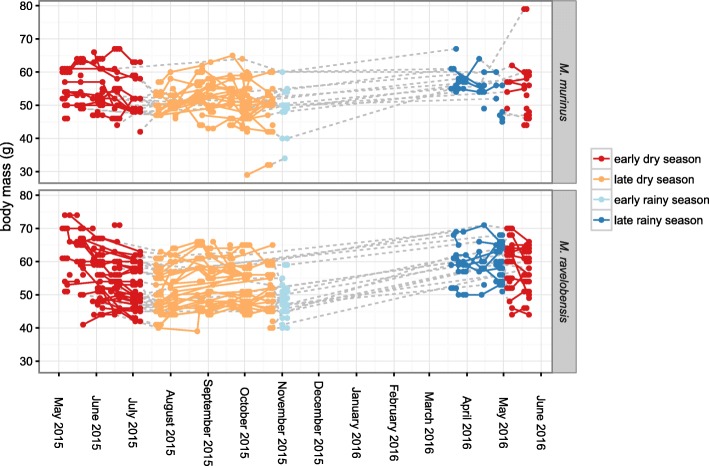
Longitudinal changes in body mass for adult, non-pregnant *M. murinus* and *M. ravelobensis* over the course of the study period. Data from April 2015 were not available

A mean decrease of 5.24 ± 0.8 g in body mass was observed in adult mouse lemurs (*n* = 153) between the early and the late dry season. From the early rainy season 2015 to the late rainy season 2016, body mass increased by an average of 7.54 ± 0.8 g (Fig. 8, Table 3). Both variations proved to be significant (*P* < 0.001). Body mass showed a statistically significant decreasing tendency of 0.071 ± 0.013 g/day (i.e. an average loss of 1 g every 14 days) within the early dry season 2015, while this pattern was reversed over the course of the late dry season with a mean daily weight gain of 0.028 ± 0.007 g. A statistically significant difference was observed between the two study species (*P* = 0.0286), with *M. ravelobensis* (*n* = 90) being generally slightly heavier than *M. murinus* (*n* = 63), but no sex-specific differences were found. Separate analyses of body mass variations for each species revealed more pronounced changes between as well as within seasons in *M. ravelobensis* than *M. murinus* (Fig. 8, Additional file 1: Table S1).

**Table 3:** Results of the linear-mixed effects model, including *post-hoc* results of simultaneous tests for general linear hypotheses for body mass differences between and within seasons

Factor	Estimate	SE	*P*-value	Effect on body mass
Species	3.699	1.474	0.029^*^	*M. ravelobensis* > *M. murinus*
Sex	-1.024	1.756	0.561	
Differences between seasons^a^				
EDS16 *vs* EDS15	5.458	1.021	<0.001^b*^	EDS16 > EDS15
LDS15 *vs* EDS15	-5.239	0.803	<0.001^b*^	EDS15 > LDS15
ERS15 *vs* EDS15	-3.406	0.959	0.004^b*^	EDS15 > ERS15
LRS16 *vs* EDS15	4.032	1.098	0.003^b*^	LRS16 > EDS15
LDS15 *vs* EDS16	-10.697	0.92	<0.001^b*^	EDS16 > LDS15
ERS15 *vs* EDS16	-8.864	1.017	<0.001^b*^	EDS16 > ERS15
LRS16 *vs* EDS16	-1.426	1.111	0.698^b^	
ERS15 *vs* LDS15	1.832	0.843	0.191^b^	
LRS16 *vs* LDS15	9.271	1.014	<0.001^b*^	LRS16 > LDS15
LRS16 *vs* ERS15	7.438	1.097	<0.001^b*^	LRS16 > ERS15
Variations within season^c^				
Early dry season 2015	-0.071	0.013	<0.001^b*^	Decrease in body mass
Late dry season 2015	0.028	0.007	<0.001^b*^	Increase in body mass
Early rainy season 2015	0.014	0.095	1^b^	
Late rainy season 2016	0.008	0.025	0.999^b^	
Early dry season 2016	-0.061	0.036	0.384^b^	

^a^Differences between mean bodyweights at the beginning of seasons

^b^Adjusted *P*-value

^c^Estimated regression slopes of body weight, depending on time within season

^*^P < 0.05

*Abbreviations*: *EDS15* early dry season 2015, *LDS15* late dry season 2015, *ERS15* early rainy season 2015, *LRS16* late rainy season 2016, *EDS16* early dry season 2016, *SE* standard deviation

## Discussion

A number of studies have investigated ectoparasites of Malagasy primates and have found lemurs hosting ticks (*Ixodes lemuris*, *Haemaphysalis lemuris* and *H. simplex*), mites (e.g. Psoroptidae, Laelaptidae), lice (sucking lice: *Lemurpediculus* spp., *Phthirpediculus* sp.; chewing lice: *Trichophilopterus babakotophilus*), endemic hippoboscid flies (*Allobosca crassipes* and *Parabosca alata*) and one introduced flea species (*Echidnophaga gallinacean*) (for the Cheirogaleidae summarized in Zohdy et al. [63]). In this study, individuals of *Microcebus murinus* and *M. ravelobensis* were infested with a haemaphysaline tick species (*Haemaphysalis* sp. “microcebi”), lice species (*Lemurpediculus* sp.) and two mite species belonging to the families of Trombiculidae and Laelaptidae. Ectoparasite infestation was influenced by temporal (sampling month and year) and potentially socioecological factors, since species differences in ectoparasite infestation might be due to divergences in sociality and the choice of sleeping sites. The findings for the different ectoparasite taxa are discussed in succession.

### Ticks

A great effort was undertaken by Harry Hoogstraal in the middle of the last century to characterize the haemaphysaline fauna of Madagascar. The original descriptions of *H. elongata* and *H. simplex* [64], *H. obtusa* [65] and *H. hoodi madagascariensis* [66] were complemented by the description of nine new species (*H. anoplos*, *H. eupleres*, *H. fossae*, *H. lemuris*, *H. nesomys*, *H. simplicima*, *H. subelongata*, *H. theilerae* and *H. tiptoni*) [39–43]. More recent studies [20–22, 67] identified collected tick specimens based on these descriptions and complementary *cox*1 gene sequences were generated for *H. lemuris* by Blanco et al. [68]. Assuming a correct classification in this previous study, our collected tick specimens showed distinct genetic differences (only 85% nucleotide identity with published *H. lemuris* sequences obtained from a *M. rufus* host), even though morphological characteristics visualized by light microscopy resembled the description of *H. lemuris*. Recently, a new species of the genus *Ixodes*, *I. inopinatus*, has been described, that can be differentiated from the similar *I. ricinus* only by combination of critical characteristics [69]. Similar subtle differences may be present in *H. lemuris* and the tick specimens collected in this study. Taking into account that the adult tick is considered the diagnostic stage, i.e. identification to species level is best achieved with adults [70], and that we only collected two adult male specimens, further sample collection including adult females will be necessary to delineate a new species and to describe it properly. Based on genetic sequence comparison of ticks collected in the present study, we consider *Haemaphysalis* sp. “microcebi” a putative new species. Our results emphasize the limitations of mere morphological differentiation of arthropod species and highlight the informative value of supplementary molecular genetic analyses, as demonstrated for *I. inopinatus* [69]. However, genetic comparison was only based on *cox*1 sequences as other genetic data, e.g. *18S* rRNA gene sequences, are not available for *H. lemuris*. Thus, generation or release of additional sequences is desirable to assess genetic relationships more reliably.

The seasonal distribution of the different developmental stages of *Haemaphysalis* sp. “microcebi” can be explained by a univoltine life-cycle, i.e. only one generation of ticks per year, as proposed for *H. lemuris* [71]. *Haemaphysalis* spp. are known to be three-host ticks, needing a suitable new host for each developmental stage. Larvae may infest mouse lemurs in the early dry season (May to July) and develop into nymphs which can be found feeding on mouse lemurs into the rainy season. Adult haemaphysaline ticks may infest larger-bodied hosts, such as larger lemurs, tenrecs and introduced species, in the rainy season. However, the detection of two adult male ticks in our study suggests a modification for *Haemaphysalis* sp. “microcebi” with respect to the proposed life-cycle model for *H. lemuris* [71] by adding mouse lemurs as potential hosts also for adult ticks in the rainy season. Detection of adult haemaphysaline ticks on *Lepilemur edwardsi* [72] in the rainy season supports the predicted univoltine life-cycle and implies a vector potential for disease transmission between endemic lemur species.

Tick infestation is shaped on the one hand by the chance to encounter questing ticks and on the other hand by the host’s ability to effectively dispose of encountered parasites. *Microcebus murinus* and *M. ravelobensis* are both known to descend to the forest floor while foraging [28] and are thus equally exposed to questing ticks, which are usually found less than one meter above ground [73]. Tick encounters may be lower during the rainy season when other food sources, such as fruits and nectar in the higher forest layers, become available, reducing the need to descend to the floor [29]. The peak in tick infestation in the dry season and the decrease in the rainy season in our study is consistent with such seasonal changes in the feeding ecology of the two mouse lemur species.

Disposal of arthropods can be achieved by means of self-grooming or allogrooming. Lemurs have a particular tooth arrangement in the lower jaw, with finely spaced incisors and canine teeth facing forward, forming a toothcomb and thus providing an effective tool for grooming [74]. The vast majority of ticks in our study, however, were found attached to the animals’ ears, a part of the body that cannot be reached by teeth for self-grooming. The higher degree of sociality at the sleeping site of *M. ravelobensis*, and corresponding higher allogrooming rates, can contribute to tick removal and might explain the significantly lower infestation rate in this host species. The same positive effects of sociality on parasite removal were expected for female *M. murinus*, who are known to sleep in groups, but no significant difference was detected between the sexes. Exemplary sleeping site data of ten female *M. murinus* (27.78% of all caught female *M. murinus*) revealed that during our study, in contrast to previous findings [33], female *M. murinus* were found predominantly sleeping solitarily (unpublished results). If these findings represent the overall sleeping patterns of female *M. murinus* in the studied population during our research period, this could well explain the species differences observed in tick infestation in this study.

### Lice

Sucking lice are obligate, permanent ectoparasites, which have evolved special morphological adaptations to their life on the mammalian host, such as tibio-tarsal claws for attachment to the hair and piercing mouthparts to penetrate the skin and feed directly from the blood vessel [75]. Being entirely dependent on the vertebrate host for survival, sucking lice have a very intimate host-parasite relationship and depend on direct body contact for transmission from one individual to another, as the time they can spend off a host is limited. This may be the underlying cause for the higher incidence and more rapid spread of lice in the group-sleeping *M. ravelobensis* as observed in this study. The intimate host-parasite relationship makes sucking lice also more susceptible to varying conditions of the host. Zohdy et al. [76] registered an increase in the louse population prior to the breeding period of the host *M. rufus* in eastern Madagascar, suggesting that reproduction of *L. verruculosus* may be triggered by an increase of host sex hormones in the imbibed blood. Frequent social interactions during the mating season increase transmission possibilities for sucking lice and the synchronization of reproductive activity may thus prove favorable for the parasite. We also observed an increase in lice infestation together with the onset of testis development, starting in July. However, we would not ascribe this phenomenon solely to the influence of sex hormones. In sheep, it is very well known that animals under stress and in poor body condition carry the heaviest lice infestations [77]. For the mouse lemurs in the Ankarafantsika National Park, the dry season constitutes a period of food scarcity and corresponding nutritional stress, which was reflected in a significant decrease in body weight of our study population. The availability of high quality food resources such as fruits, flowers and nectar, declines over the course of the dry season, until an increase in flowering is again observed in September [28, 29]. The observed variations in body mass correspond to the seasonal changes in the availability of high-quality food resources. Thus, it seems likely that the increase in lice infestations is due to the additive effects of internal (hormonal activity, mating season) and external stressors (nutritional stress). Since weight loss was more prominent in *M. ravelobensis*, whereas reproductive activity was observed for both studied host species equally, external factors like food abundance, i.e. nutritive stress, and social factors like sleeping-group composition/body contact may be the main parameters influencing lice infestation dynamics of *M. murinus* and *M. ravelobensis*.

Feeding on several different hosts makes lice also potential vectors for disease transmission. Sucking lice have been identified as vectors for pathogens such as poxvirus in pigs [78], *Mycoplasma* in rats and mice [70] or *Borrelia recurrentis* causing epidemic relapsing fever in humans [79]. Also, parasites may be transmitted *via* sucking lice as shown for the seal louse *Echinophthirius horridus* that has been identified as an intermediate host for microfilariae of the seal heartworm *Acanthocheilonema spirocauda* (formerly called *Dipetalonema spirocauda*) [80]. As ectoparasites of *M. murinus* and *M. ravelobensis*, *Lemurpediculus* sp. may play a role in the transmission of blood-borne pathogens within one species as well as between the two *Microcebus* species. Apart from this potential role as a vector, sucking lice can also have a harmful effect on the host by inducing anemia due to blood loss that may result in weakness or stunted growth, especially in young animals. There is a need for additional investigations for a more precise taxonomic classification of the collected sucking lice specimens. A comparative study, including morphological and genetic analysis, may help to complement the picture of interrelations between sucking lice and mouse lemurs.

### Mites

This study is the first report of chigger mites (family Trombiculidae) in a lemur species. Trombiculid mites occur throughout the world and more than 3000 species have been identified [81]; however, little is known about the chigger fauna of Madagascar. Thus, the collected specimens, isolated from skin scrapings of *M. murinus* and *M. ravelobensis*, could not be identified to the species level, but genetic analysis confirmed assignment to the family Trombiculidae. In most chigger species, the larval stage, which is the only parasitic stage of this arthropod, feeding on lymph or tissue fluid from an animal’s skin, shows large seasonal fluctuations. While temperature has been identified as the limiting factor in temperate regions, the amount of precipitation seems to be of greater influence on chigger mite abundance in tropical areas [82]. In the present study, only two incidences of skin alterations caused by trombiculid mites were observed in September 2015, and no trombiculid activity was registered in October and November 2015, when relative humidity was below 55%. These observations support the influence of humidity on chiggers’ development. Chigger mite infestations were significantly more often encountered in the dry season, but the overall low frequency of Trombiculidae gen. sp. on both mouse lemur hosts did not allow a more detailed statistical analysis, that may enable differentiation between early and late dry season or correlation with climatic parameters. Low numbers of chigger mite infestations observed in the hot and humid month of April 2015 and March 2016 could be attributed to the lower sample size (April 15, *n* = 15; March 16, *n* = 22), as the high food abundance in the forest at the end of the rainy season had a negative impact on trapping success. The results for these months should therefore be interpreted cautiously.

Many chigger species show a preference to a certain area of the host’s body. For the collected Trombiculidae gen. sp. the predilection site was the face, especially around the eyes and the lateral snout, where the animals are not able to groom themselves by means of their toothcomb. Skin alterations due to trombiculid larvae were significantly more often observed in *M. murinus*. As discussed above regarding tick infestations, the higher degree of sociality at the sleeping site of *M. ravelobensis* which should coincide with higher allogrooming rates, may have increased disposal of trombiculid mites.

The second mite species collected from *M. murinus* and *M. ravelobensis* in this study could again not be identified to species level; however, genetic analyses assigned the collected specimens to the family Laelaptidae. Laelapid mites have been described as facultative or obligate parasites in nests of mammals and *Androlaelaps* mites, a genus of lealaptid mites known as parasites of Malagasy lemurs, are characterized as nidiculous, polyphagous opportunists with varying degrees of parasitic feeding [83]. This matches our findings of laelapid mites occurring only occasionally (*n* = 19) and with the exception of two incidences exclusively on *M. murinus*. The frequent use of tree holes as daytime sleeping sites by this host species presents an ideal setting to maintain the mites’ life-cycle. The mouse lemurs’ sleeping site provides a rich food supply, such as scabs and excretions, and the animal itself facilitates dispersal to different sleeping sites. The parasitism by laelapid mites in mouse lemurs may, however, be underestimated, given that these nidiculous parasites only spend a limited time on the host and the detection rate on the body of the mouse lemurs does therefore not necessarily reflect the situation at the host’s sleeping site. Invasive sampling of the sleeping sites would be necessary to obtain a more comprehensive picture on Laelaptidae gen. sp. prevalence. However, we decided against this approach to avoid disturbance of the study habitat and following impairment of long-term data collection.

## Conclusions

The two mouse lemur species *M. murinus* and *M. ravelobensis* were found to host haemaphysaline ticks, lice and mites in the Ankarafantsika National Park of northwestern Madagascar. The probability of ectoparasite infestation was influenced by temporal (month, year) parameters, and by the host species, which could potentially be attributed to differences in the socioecology of the two host species. The results of this longitudinal study give a more comprehensive picture of the complex dynamics between environment and host-parasite interactions. The seasonal variations in ectoparasite prevalence need to be considered when interpreting ectoparasite data of a particular period in future studies. This is particularly relevant, as Madagascar is known for extreme climatic variations and unpredictable weather conditions between and within seasons and years [84] and environmental stressors therefore need to be evaluated thoroughly. The detection of the same ectoparasite species in two closely related sympatric hosts indicates a potential cross-species pathway for pathogen transmission. While ectoparasites themselves may have a negative effect on individuals through irritation, impairment of the natural barrier function of the skin and anemia due to blood-feeding, they may also represent potential vectors for disease transmission between individuals. Further studies will be needed to investigate possible vector-borne pathogens circulating in the studied *M. murinus* and *M. ravelobensis* populations, which might also pose a risk to other endemic species as well as humans and livestock.

## Additional file

Additional file 1: **Table S1.** *Post-hoc* results of simultaneous tests for general linear hypotheses for body mass differences between and within seasons for *M. murinus* and *M. ravelobensis*. (DOCX 25 kb)
