# Supplementary material for: Ectoparasite communities of small-bodied Malagasy primates: seasonal and socioecological influences on tick, mite and lice infestation of Microcebus murinus and M. ravelobensis in northwestern Madagascar
Source: Parasit Vectors. 2018 Aug 8;11:459. doi: 10.1186/s13071-018-3034-y (PMC6083607; doi:10.1186/s13071-018-3034-y)
Supplement: Supplementary file 1 — Table S1. Post-hoc results of simultaneous tests for general linear hypotheses for body mass differences between and within seasons for M. murinus and M. ravelobensis. (DOCX 25 kb) [file 13071_2018_3034_MOESM1_ESM.docx]

**Additional file 1: Table S1.** *Post-hoc* results of simultaneous tests for general linear hypotheses for body mass differences between and within seasons for *M. murinus* and *M. ravelobensis*

| **Factor** | **Estimate** | **Std. Error** | **Adjusted *P*‑values** | **Effect on body mass** |
| --- | --- | --- | --- | --- |
| *Microcebus murinus*  Difference between seasons^a^ | |  |  |  |
| EDS16 *vs* EDS15 | 6.000 | 1.837 | 0.011* | EDS16 > EDS15 |
| LDS15 *vs* EDS15 | -2.800 | 1.324 | 0.214 |  |
| ERS15 *vs* EDS15 | -1.415 | 1.637 | 0.908 |  |
| LRS16 *vs* EDS15 | 4.010 | 1.709 | 0.132 |  |
| LDS15 *vs* EDS16 | -8.801 | 1.707 | < 0.001* | EDS16 > LDS15 |
| ERS15 *vs* EDS16 | -7.415 | 1.921 | 0.001* | EDS16 > ERS15 |
| LRS16 *vs* EDS16 | -1.990 | 1.972 | 0.848 |  |
| ERS15 *vs* LDS15 | 1.386 | 1.477 | 0.879 |  |
| LRS16 *vs* LDS15 | 6.810 | 1.601 | < 0.001* | LRS16 > LDS15 |
| LRS16 *vs* ERS15 | 5.425 | 1.780 | 0.021* | LRS16 > ERS15 |
| Variations within seasons^b^ | |  |  |  |
| Early dry season 2015 | -0.054 | 0.023 | 0.081 |  |
| Late dry season 2015 | 0.023 | 0.012 | 0.290 |  |
| Early rainy season 2015 | 0.020 | 0.202 | 1.000 |  |
| Late rainy season 2016 | -0.020 | 0.043 | 0.995 |  |
| Early dry season 2016 | -0.024 | 0.079 | 0.999 |  |
| *Microcebus ravelobensis*  Difference between seasons^a^ | |  |  |  |
| EDS16 *vs* EDS15 | 5.086 | 1.193 | < 0.001* | EDS16 > EDS15 |
| LDS15 *vs* EDS15 | -6.730 | 0.962 | < 0.001* | EDS15 > LDS15 |
| ERS15 *vs* EDS15 | -4.604 | 1.132 | < 0.001* | EDS15 > ERS15 |
| LRS16 *vs* EDS15 | 4.615 | 1.377 | 0.008* | LRS16 > EDS15 |
| LDS15 *vs* EDS16 | -11.816 | 1.051 | < 0.001* | EDS16 > LDS15 |
| ERS15 *vs* EDS16 | -9.690 | 1.142 | < 0.001* | EDS16 > ERS15 |
| LRS16 *vs* EDS16 | -0.471 | 1.309 | 0.996 |  |
| ERS15 *vs* LDS15 | 2.126 | 0.974 | 0.187 |  |
| LRS16 *vs* LDS15 | 11.346 | 1.261 | < 0.001* | LRS16 > LDS15 |
| LRS16 *vs* ERS15 | 9.219 | 1.347 | < 0.001* | LRS16 > ERS15 |
| Variations within seasons^b^ | |  |  |  |
| Early dry season 2015 | -0.085 | 0.015 | < 0.001* | Decrease in body mass |
| Late dry season 2015 | 0.029 | 0.009 | 0.004* | Increase in body mass |
| Early rainy season 2015 | 0.015 | 0.103 | 1 |  |
| Late rainy season 2016 | 0.008 | 0.029 | 1 |  |
| Early dry season 2016 | -0.076 | 0.038 | 0.222 |  |

EDS15 = early dry season 2015, LDS15 = late dry season 2015, ERS15 = early rainy season 2015, LRS16 = late rainy season 2016, EDS16 = early dry season 2016

^a^ Differences between mean body masses at the beginning of seasons

^b^ Estimated regression slopes of body masses, depending on time within season

* statistically significant (*P* < 0.05)
